# Early Point-of-Care Thromboelastometry Reduces Mortality in Patients with Severe Trauma and Risk of Transfusion: An Analysis Based on the TraumaRegister DGU^®^

**DOI:** 10.3390/jcm13144059

**Published:** 2024-07-11

**Authors:** Christoph Beyersdorf, Dan Bieler, Rolf Lefering, Sebastian Imach, Lisa Hackenberg, Erik Schiffner, Simon Thelen, Felix Lakomek, Joachim Windolf, Carina Jaekel, TraumaRegister DGU

**Affiliations:** 1 Department of Orthopedics and Trauma Surgery, Medical Faculty, University Hospital Duesseldorf, Heinrich-Heine-University Duesseldorf, 40225 Duesseldorf, Germany; christoph.beyersdorf@med.uni-duesseldorf.de (C.B.); erik.schiffner@med.uni-duesseldorf.de (E.S.); simon.thelen@med.uni-duesseldorf.de (S.T.); felix.lakomek@med.uni-duesseldorf.de (F.L.); joachim.windolf@med.uni-duesseldorf.de (J.W.); carina.jaekel@med.uni-duesseldorf.de (C.J.); 2 Department for Trauma Surgery and Orthopaedics, Reconstructive and Hand Surgery, Burn Medicine, German Armed Forces Central Hospital, 56072 Koblenz, Germany; lisa.hackenberg@gmx.de; 3 Institute for Research in Operative Medicine (IFOM), University Witten/Herdecke, 58455 Cologne, Germany; rolf.lefering@uni-wh.de; 4 Department of Trauma and Orthopedic Surgery, Cologne-Merheim Medical Center (CMMC), University Witten/Herdecke, 58455 Cologne, Germany; imachse@kliniken-koeln.de; 5 Committee on Emergency Medicine, Intensive Care and Trauma Management (Sektion NIS) of the German Trauma Society (DGU®), 10623 Berlin, Germany; support-tr@auc-online.de

**Keywords:** coagulopathy, ROTEM^®^, thromboelastometry, point-of-care, trauma, bleeding, massive transfusion

## Abstract

**Background:** Thromboelastometry like ROTEM^®^ is a point-of-care method used to assess the coagulation status of patients in a rapid manner being particularly useful in critical care settings, such as trauma, where quick and accurate assessment of coagulation can guide timely and appropriate treatment. Currently, this method is not yet comprehensively available with sparse data on its effectiveness in resuscitation rooms. The aim of this study was to assess the effect of early thromboelastometry on the probability of mass transfusions and mortality of severely injured patients. **Methods:** The TraumaRegister DGU^®^ was retrospectively analyzed for severely injured patients (2011 until 2020) with information available regarding blood transfusions and Trauma-Associated Severe Hemorrhage (TASH) score components. Patients with an estimated risk of mass transfusion >2% were included in a matched-pair analysis. Cases with and without use of ROTEM^®^ diagnostic were matched based on risk categories for mass transfusion. A total of 1722 patients with ROTEM^®^ diagnostics could be matched with a non-ROTEM^®^ patient with an identical risk category. Adult patients (≥16) admitted to a trauma center in Germany, Austria, or Switzerland with Maximum Abbreviated Injury Scale severity ≥3 were included. **Results:** A total of 83,798 trauma victims were identified after applying the inclusion and exclusion criteria. For 7740 of these patients, the use of ROTEM^®^ was documented. The mean Injury Severity Score (ISS) in patients with ROTEM^®^ was 24.3 compared to 19.7 in the non-ROTEM^®^ group. The number of mass transfusions showed no significant difference (14.9% ROTEM^®^ group vs. 13.4% non-ROTEM^®^ group, *p* = 0.45). Coagulation management agents were given significantly more often in the ROTEM^®^ subgroup. Mortality in the ROTEM^®^ group was 4.1% less than expected (estimated mortality based on RISC II 34.6% vs. observed mortality 30.5% (n = 525)). In the non-ROTEM^®^ group, observed mortality was 1.6% less than expected. Therefore, by using ROTEM^®^ analysis, the expected mortality could be reduced by 2.5% (number needed to treat (NNT) 40; SMR of ROTEM^®^ group: 1:0.88; SMR of non-ROTEM^®^ group: 1:0.96; *p* = 0.081). **Conclusions:** Hemorrhage is still one of the leading causes of death of severely injured patients in the first hours after trauma. Early thromboelastometry can lead to a more targeted coagulation management, but is not yet widely available. This study demonstrated that ROTEM^®^ was used for the more severely injured patients and that its use was associated with a less than expected mortality as well as a higher utilization of hemostatic products.

## 1. Introduction

Traumatic hemorrhage remains the leading cause of preventable deaths in injured patients with one third of multiple trauma patients presenting trauma-induced coagulopathy on hospital arrival [1,2,3]. Effective coagulation management is an important but sometimes difficult task to accomplish in the early phases following severe trauma. Management of coagulopathy starts in the pre-hospital phase with hemostatic agents such as tranexamic acid. In the in-hospital phase, most advanced trauma centers use predefined ratios of packed red blood cells (pRBCs), plasma, and platelets as well as hemostatic agents and coagulation (clotting) factors. In recent years, several scores to detect the probability for massive transfusion (MT), such as the Assessment of Blood Consumption (ABC), McLaughlin, or Trauma-Associated Severe Hemorrhage (TASH) scores, have been implemented to detect high-risk patients and potentially influence early therapeutic strategies [4,5,6,7]. The components of all scores in particular are contained in the documentation forms of the TraumaRegister DGU^®^ of the German Trauma Society (Deutsche Gesellschaft für Unfallchirurgie, DGU^®^). Only the TASH score weights data. The other scores are dichotomous.

Available for early coagulation diagnostics are standard laboratory tests like International Normalized Ratio (INR), prothrombin time (PT), platelet counts, fibrinogen level, and blood gas analysis as well as diagnostic imaging and patient physiology [2]. In recent years, more focus has been placed on point-of-care (POC) diagnostics such as rotational thromboelastometry (ROTEM^®^). This further development of the method originally described in 1948 by Hellmut Hartert allows for assessment of clot formation and degradation within minutes and thereby potentially a more targeted therapy [8]. For this purpose, a blood sample is drawn and analyzed in the resuscitation room and can be repeated if necessary.

We hypothesized that the usage of thromboelastometry in the resuscitation room can reduce the risk for MT as predicted via TASH score and decrease mortality through a more targeted therapy. MT in this study is defined as transfusion of 10 or more pRBCs.

## 2. Materials and Methods

The TraumaRegister DGU^®^ (TR-DGU^®^) was founded in 1993. The aim of this multicenter database is a pseudonymized and standardized documentation of severely injured patients.

Data are collected prospectively in four consecutive time phases from the site of the accident until discharge from hospital: (A) Pre-hospital phase, (B) Emergency room and initial surgery, (C) Intensive care unit, and (D) Discharge. The documentation includes detailed information on demographics, injury pattern, comorbidities, pre- and in-hospital management, a course on the intensive care unit, relevant laboratory findings including data on transfusion and outcome of each individual. The inclusion criterion is admission to hospital via emergency room with subsequent ICU/ICM care or reaching the hospital with vital signs and dying before admission to ICU. The infrastructure for documentation, data management, and data analysis is provided by AUC—Academy for Trauma Surgery (AUC—Akademie der Unfallchirurgie GmbH), a company affiliated to the German Trauma Society. The scientific leadership is provided by the Committee on Emergency Medicine, Intensive Care and Trauma Management (Sektion NIS) of the German Trauma Society. The participating hospitals submit their data pseudonymized into a central database via a web-based application. Scientific data analysis is approved according to a peer review procedure laid down in the publication guideline of TraumaRegister DGU^®^. The participating hospitals are primarily located in Germany (90%), but a rising number of hospitals of other countries contribute data as well (at the moment from Austria, Belgium, China, Finland, Luxembourg, Slovenia, Switzerland, The Netherlands, and the United Arab Emirates). Currently, more than 35,000 cases from almost 700 hospitals are entered into the database per year. Participation in TR-DGU^®^ is voluntary. For hospitals associated with TraumaNetzwerk DGU^®^, however, the entry of at least a basic data set is obligatory for reasons of quality assurance. The last validation of the TASH score dates back to 2010 on datasets from the 2004–2007 TR-DGU^®^ database [4]. Therefore, we analyzed the actual 2011–2020 TR-DGU^®^ database to update the TASH components.

For estimation of the risk of death in hospital, version II of the Revised Injury Severity Classification (RISC II) was used [9]. This score combines 13 different information available shortly after admission. It has been developed and validated with TR-DGU^®^ data.

### 2.1. Patients

Trauma patients primarily admitted (no transfers) to hospitals in Germany, Austria, or Switzerland in 2011–2020 who were documented with the standard form qualified for analysis. Only patients with trauma team activation and Maximum Abbreviated Injury Scale (MAIS) severity ≥3 were included; patients with MAIS 2 were included only if they required intensive care, or if they died in hospital. Furthermore, young patients <16 years of age were excluded, as were patients with missing data regarding blood transfusion or TASH components (initial blood pressure, hemoglobin, base excess).

Since 2009, ROTEM^®^ analysis is part of the standard documentation form of TR-DGU^®^. The information about the performance of a ROTEM^®^ analysis (yes/no) in the resuscitation room was available in 79% of cases; if no information was documented, it was assumed that no such analysis had been performed. The majority of hospitals (129 of 231, 56%) did not perform any ROTEM^®^ analysis during the 10 years’ time period, and another 54 hospitals performed fewer than 10 analyses.

### 2.2. Statistics

Descriptive analysis was performed with SPSS (version 24, IBM Corp., Armonk, NY, USA). Numbers of cases, percentages, means, and standard deviations (SD)s were provided. In case of skewed distributions, median with quartiles were reported. The chi-squared test was used for categorical variables, the Mann–Whitney *U* test was used for metric variables. A *p*-value < 0.05 was considered statistically significant.

After initial assessment of the precision of the existing TASH score, an improved formula for prediction of MT was developed using logistic regression analysis. Based on this improved prediction, categories of similar risk were created. In cases with a risk for MT of at least 2%, cases with and without use of ROTEM^®^ diagnostics were matched based on the identical risk category. This resulted in a subset of 1722 patients from each subgroup (ROTEM^®^ and non-ROTEM^®^).

The study was performed in accordance with the publication guideline of the TraumaRegister DGU^®^ and is registered as TR-DGU^®^ Project ID 2021–028. Since the study was a retrospective anonymized analysis, ethical approval was not required according to the regulations of the responsible regional medical association. The authors had no access to information that could identify individual participants during or after data collection. Anonymous data were accessed on 8 December 2021.

## 3. Results

**Figure 1:**
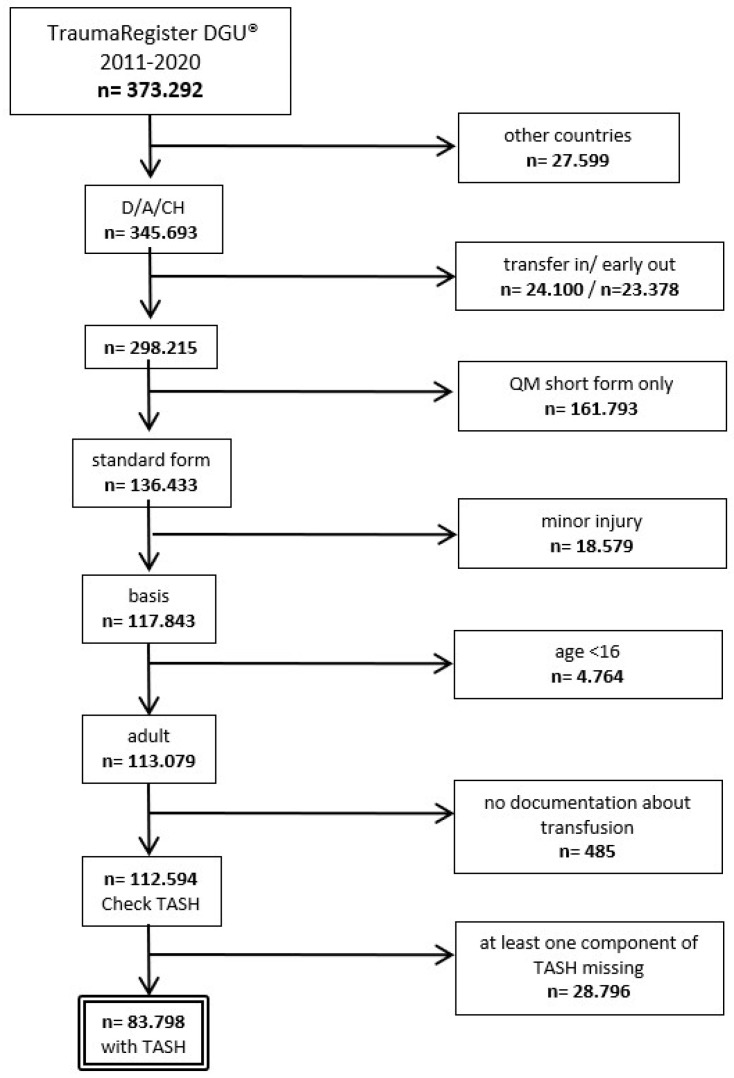
Cohort identification. Legend: TASH: Trauma-Associated Severe Hemorrhage; D: Germany, A: Austria, CH: Switzerland.

Between 2011 and 2020, 83,798 trauma victims were identified after applying the above-stated inclusion and exclusion criteria (Figure 1). In this group, 9220 patients (11.0%) received blood in the emergency room or during the initial operative phase until ICU admission. A total of 1461 patients (1.7%) received 10 or more units of packed red blood cells (pRBC, MT in that period).

### 3.1. TASH Validation and General Data

Table 1 shows the prevalence of the TASH components, and the frequency of MT for each condition observed. The mean TASH score was 4.0 (SD 4.1, median 2, IQR 1–5). This corresponds to an expected rate of 3.8% for MT. This was twice as high as observed.

Therefore, a logistic regression analysis was conducted with MT as dependent variable (see Table S1 in the Supplementary Materials). In contrast to the original model, the blood pressure category <100 was further split into <90 and 90–99. The results of the regression analysis were used to derive an optimized estimation for risk of MT in the actual collective.

**Figure 2:**
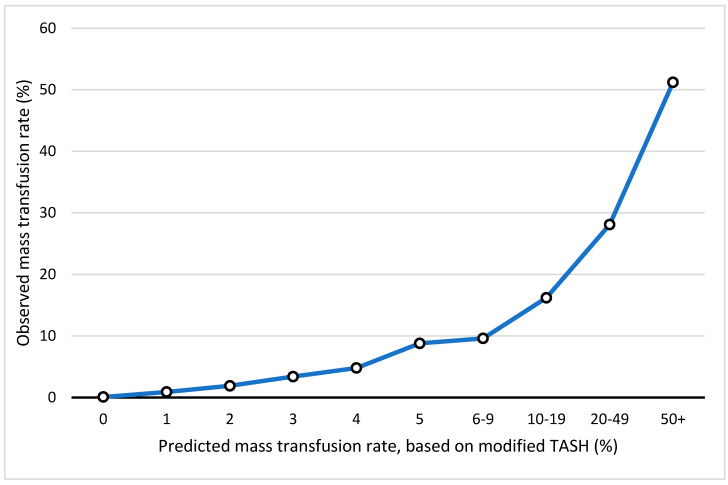
Comparison of the predicted massive transfusion rate based on modified TASH score with the actual documented massive transfusion rate for each TASH score. Legend: TASH: Trauma-Associated Severe Hemorrhage.

To analyze whether the optimized TASH score provides an accurate assessment for the probability of MT, we compared the predicted rate with the actually observed rate of MT in our dataset (Figure 2).

### 3.2. ROTEM^®^ Subpopulation

For 7740 patients, use of the ROTEM^®^ analysis in the resuscitation room was documented, which represents 9.2% of the study cohort.

**Figure 3:**
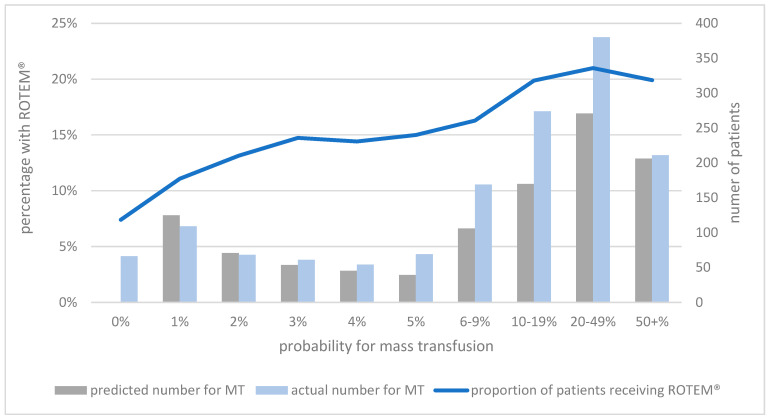
The different probabilities for massive transfusion as predicted via TASH score and the respective proportion of patients receiving ROTEM^®^ analysis as well as the predicted and actual number of patients with massive transfusions.

The mean age was 50.2 years. A total of 26.5% of this subpopulation were female and 73.5% male. The proportion of patients receiving a ROTEM^®^ analysis increased with increasing probability for MT. Moreover, with increasing probability for MT and use of ROTEM^®^, the difference between predicted and observed numbers of patients with MT increased (Figure 3).

Patients who were included in a ROTEM^®^ analysis seemed to be more severely injured. To this extent, the mean Injury Severity Score (ISS) in patients with ROTEM^®^ was 24.3 compared to 19.7 (*p* < 0.001) in patients without this analysis. Similarly, the mean number of days spent on ICU was higher (9.9 vs. 7.0 days, *p* < 0.001), as was mean length of stay in hospital (22.0 vs. 17.0 days). Upon arrival in the resuscitation room, more patients in the ROTEM^®^ subpopulation showed indications for shock with systolic blood pressure (SBP) values of ≤90 mmHg (15.8% vs. 8.8%). Accordingly, this group received more often catecholamine therapy (33.2% vs. 17.4%) as well as packed red blood cells (pRBCs; 22.8% vs. 9.8%) in the resuscitation room.

Moreover, plasmatic coagulation seemed to be more impaired in the ROTEM^®^ subpopulation. Average INR values were 1.23 compared to 1.19 in patients without this analysis (see Table 2).

### 3.3. Patterns of Injury

The predominant causes for admission to the resuscitation room in the ROTEM^®^ subpopulation were car and motorcycle accidents as well as high falls. Compared to the non-ROTEM^®^ population, the proportion of penetrating injuries was considerably higher in the ROTEM^®^ population.

Looking at the AIS scores for head, thorax, abdomen, and extremities, the ROTEM^®^ subpopulation exhibits a higher proportion of individuals with a score of ≥3 in each category (see Table 2).

### 3.4. Coagulation Management and Outcome

To further assess whether the conduction of a ROTEM^®^ analysis had an effect on coagulation management in the resuscitation room, we performed a matched pair analysis of patients who received ROTEM^®^ and those who did not. Only patients with an estimated risk of MT > 2% (n = 10,446) were included in this sub-analysis. A total of 1722 patients received a ROTEM^®^ diagnostic (16.5%), and all these cases could be matched with a non-ROTEM^®^ patient with identical risk category.

**Figure 4:**
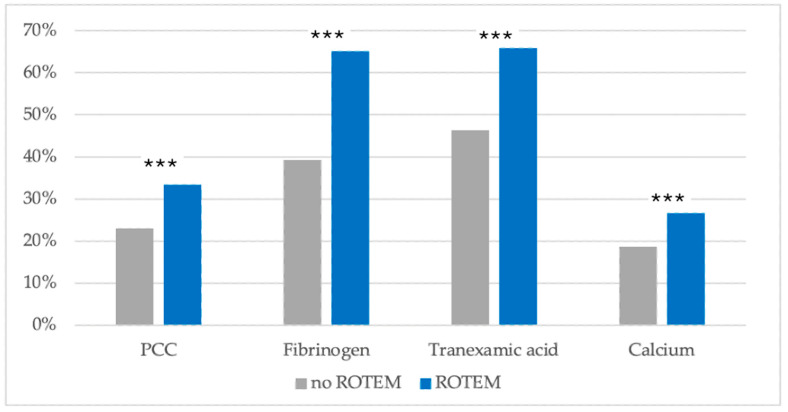
Comparison of the usage of indicated coagulation management agents in the ROTEM^®^ and non-ROTEM^®^ subgroups. PCC: Prothrombin complex concentrate. Data are illustrated after matched pair analysis with the TASH-based risk for massive transfusion as a matching criterion. ***: *p* < 0.001 (in comparison to the non-ROTEM^®^ counterpart). N = 1722 for each subgroup. Legend: ROTEM^®^: rotational thromboelastometry; PCC: Prothrombin complex concentrate.

In the ROTEM^®^ subgroup, prothrombin complex concentrate (PCC), fibrinogen, tranexamic acid, and calcium were all given significantly more often (Figure 4).

Of note, pRBCs were given significantly more often in the ROTEM^®^ subgroup (64.6% vs. 54.9%, *p* < 0.001), while the number of MTs remained approximately equal (14.9% in the ROTEM^®^ group vs. 13.4% in the non-ROTEM^®^ group, *p* = 0.45). The use of FFP transfusion was also similar (33.3% vs. 33.0%, *p* = 0.72).

**Figure 5:**
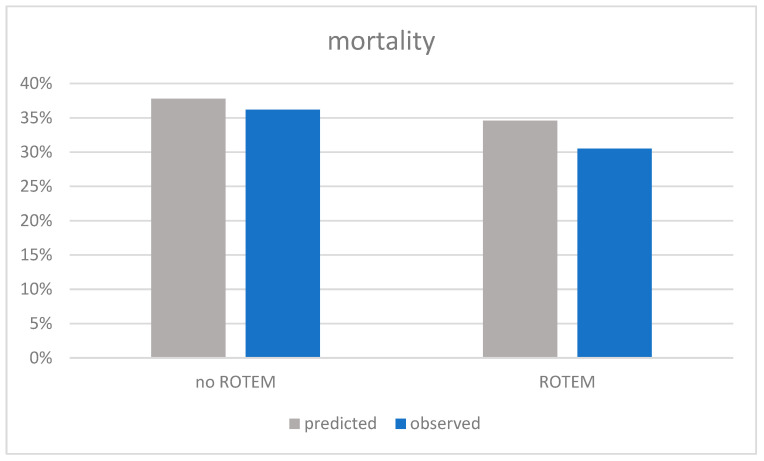
Comparison of the observed mortality and the predicted mortality as conducted via RISC-II-score. Legend: ROTEM^®^: rotational thromboelastometry; RISCII: Revised Injury Severity Classification, version II.

The estimated mortality based on RISC II was 34.6% in the ROTEM^®^ group and 37.8% in the non-ROTEM^®^ group. In contrast, the observed mortality was 30.5% in the ROTEM^®^ group (n = 525) and 36.2% in the non-ROTEM^®^ group (n = 624; Figure 5). Therefore, mortality in the ROTEM^®^ group was 4.1% less than expected. In the non-ROTEM^®^ group, observed mortality was 1.6% less than expected. Thus, the usage of ROTEM^®^ analysis correlates with a reduction in expected mortality by 2.5% (Number needed to treat (NNT) 40; SMR ROTEM^®^ group: 1:0.88; SMR non-ROTEM^®^ group: 1:0.96; *p* = 0.081).

## 4. Discussion

In this study, the use of ROTEM^®^ in the resuscitation room correlates with a reduction in expected mortality of severely injured patients by 2.5% (NNT 40).

The utilization of point-of-care diagnostics such as rotational thromboelastometry (ROTEM^®^) for coagulation management of trauma patients attracted growing interest in recent years. A differentiated analysis of coagulation parameters such as clot formation kinetics can be made, which can lead to a more targeted therapy. Traumatic coagulation disorders directly correlate with mortality of severely injured patients and are known to be present in about 25% of severely injured patients in the resuscitation room [10,11]. Therefore, early and effective management of trauma-associated coagulopathy is inevitable. Conventional laboratory tests such as INR, aPTT, and hemoglobin values represent the basic diagnostics besides clinical parameters but are not directly available as a POC diagnostics. There are however several limitations to these standard laboratory tests in trauma patients. For instance, in presence of hyperfibrinolysis or fibrinogen deficiency, international normalized ratio (INR) and aPTT are not adequately assessable [11]. Hyperfibrinolysis however is present in about 8% of trauma patients and is associated with excess mortality. Viscoelastic POC methods such as ROTEM^®^ allow for differentiated analysis of clot formation kinetics, clot strength, and fibrinolysis as well as contribution of platelets to clot-forming [11,12]. ROTEM^®^ analysis has been shown to reduce the rate of perioperative blood transfusions in cardiac and hip surgery [13,14,15,16]. This reduction could not be observed in this group of severely injured individuals. However, patients in this study are also only assessed after the accident has already occurred. Unlike in the operating room, coagulation cannot be optimized before any potential bleeding.

This study’s aim was to evaluate whether implementation of ROTEM^®^ in the resuscitation room can reduce the risk for MT as predicted via TASH score and decrease mortality through a more targeted therapy.

For only 9% of our initial study cohort, information was given about the use of ROTEM^®^. In general, thromboelastometry seems to be conducted more frequently in more severely injured patients. To this extent, the proportion of patients receiving ROTEM^®^ investigation increased with increasing probability for MT as predicted via TASH score. Also, patients with higher ISS scores received ROTEM^®^ analysis more frequently. Wake et al. recently also observed a higher proportion of major trauma patients with longer ICU stays receiving ROTEM^®^ blood tests in their observational study after implementing thromboelastometry in their trauma center [17]. This suggests a lack of routinely implemented ROTEM^®^ in severe trauma patients.

Coagulation management agents, such as fibrinogen, PCC, calcium or tranexamic acid were administered significantly more often in patients, that have received ROTEM^®^ analysis beforehand. Partially, this may be attributed to the more severely injured and therefore probably more extensively bleeding ROTEM^®^ subpopulation. It seems very likely though, that conduction of ROTEM^®^ analysis also leads to a more targeted and extensive use of these agents [18,19]. A recent study from Australia also found a significant increase in fibrinogen administration after implementation of a ROTEM^®^ protocol for patients requiring ICU admission [20]. The substitution of calcium, however, is not a direct consequence of a ROTEM^®^ analysis. Therefore, it can be assumed that there is a general sensitization towards coagulation optimization.

In addition to the severity of the injury, a longer pre-hospital phase from the injury to the hospital can be discussed as another reason for the higher blood loss in the ROTEM^®^ population. In Table 2, we show that the ROTEM^®^ population has a slightly longer pre-hospital phase (though only by an average of 4 min). However, more fluid was administered pre-hospital in this group.

The probability for MT was calculated via TASH score, as described in the last update of the score in 2010 [4]. Logistic regression analysis of each TASH component showed a positive association of each TASH component with the probability for MT. For even better correlation of the score in our population, we inserted a blood pressure interval between 90 and 100 mmHg systolic pressure, which was not originally included in the TASH score. Comparison between predicted and observed probability for MT showed comparable values.

Furthermore, in the matched pair analysis of the ROTEM^®^ subpopulation MT in the ROTEM^®^ subpopulation were administered 1.6% more frequently than expected via TASH score (14.9% vs. 13.3%, *p* = 0.450; non-ROTEM^®^: 13.4% actual MT, 13.3% expected MT via TASH score). Considering the higher severity of injury in the ROTEM^®^ subpopulation, a positive effect on occurrence of MT after thromboelastometry can be assumed. Multiple studies in recent years demonstrated the ability of early thromboelastometry to predict MT in trauma patients [21,22,23]. There also seems to be a correlation between more severely deviating ROTEM^®^ parameters and mortality [24,25]. To the best of our knowledge there are no conclusive data so far on a positive effect of conducting ROTEM^®^ on mortality. Our study shows that usage of ROTEM^®^ analysis correlates with a reduction in expected mortality by 2.5%, which justifies considering ROTEM^®^ in early resuscitation in our opinion. Although this difference did not reach statistical significance in the present cohort, a positive trend towards reduction in mortality by using ROTEM^®^ analysis in the resuscitation room can be assumed. Further prospective studies are needed to confirm this assumption.

## 5. Conclusions

This study shows that ROTEM^®^ was used for the more severely injured patients and that its use was associated with a less than expected mortality (NNT = 40) as well as a higher utilization of hemostatic products. So far however, it is not broadly available or used in resuscitation rooms. In this study, we demonstrated a correlation between ROTEM^®^ analysis and a lower-than-predicted mortality in severely injured patients.

Because of the relatively sparse information given about the usage of ROTEM^®^ analysis in the TraumaNetzwerk hospitals, further data about the individual ROTEM^®^ parameters should be collected. Subsequently, the development of an algorithm regarding the usage of ROTEM^®^ and the actions following individual parameters seems useful in order to further lower hemorrhage-associated mortality in trauma.

## 6. Limitations

This study was designed as a retrospective analysis, which has well-known limitations. Prospective follow-up studies are needed to confirm these findings. The ROTEM^®^ group in this study was more severely injured than the non-ROTEM^®^ group, which may partially limit the comparability. The data available in the TraumaRegister only allow conclusions to be drawn as to whether ROTEM^®^ was used or not. It is not known when exactly during the resuscitation phase it was used or what conclusions were drawn regarding management. Furthermore, there is no information available about the resources of the hospitals that used ROTEM^®^. It may be assumed that more patients in the ROTEM^®^ group come from larger hospitals than the patients in the non-ROTEM^®^ group. In our matched-pair analysis, however, a difference in expected and observed mortality was observed within the ROTEM^®^ group. ROTEM^®^ was associated with a higher use of hemostatic products. Since these patients also had a reduced mortality rate, the increased use of hemostatic products may be partly attributed to a longer survival time.

## Figures and Tables

**Table 1:** Comparison of the prevalence of the individual TASH components and the frequency of massive transfusion for each condition.

TASH Component	Value	TASH Points	Prevalence	Patients with MT (Percentage of Prevalence)
Hemoglobin (g/dL)	<7	8	1535 (1.8%)	366 (23.8%)
	<9	6	3430 (4.1%)	370 (10.8%)
	<10	4	3431 (4.1%)	188 (5.5%)
	<11	3	5662 (6.8%)	169 (3.0%)
	<12	2	9068 (10.8%)	154 (1.7%)
	12+	0	60,671 (72.4%)	214 (0.4%)
Base excess (mmol/L)	<−10	4	4125 (4.9%)	588 (14.3%)
	<−6	3	6086 (7.3%)	328 (5.4%)
	<−2	1	21,818 (26.0%)	346 (1.6%)
	−2+	0	51,769 (61.8%)	199 (0.4%)
Systolic blood pressure (mmHg)	<90	*	6106 (7.3%)	716 (11.7%)
	<100	4	3758 (4.5%)	161 (4.3%)
	<120	1	14,116 (16.8%)	266 (1.9%)
	120+	0	59,818 (71.4%)	318 (0.5%)
Heart rate (b/min)	>120	2	5029 (6.0%)	396 (7.9%)
	≤120	0	78,769 (94.0%)	1065 (1.4%)
FAST positive (AIS 3+ organ injury)	yes	3	8385 (10.0%)	789 (9.4%)
	no	0	75,413 (90.0%)	672 (0.9%)
Femur fracture	yes	3	13,260 (15.8%)	601 (4.5%)
	no	0	70,583 (84.2%)	860 (1.2%)
Unstable pelvic fracture	yes	6	1567 (1.9%)	323 (20.6%)
	no	0	82,231 (98.1%)	1138 (1.4%)
Gender	M	1	59,890 (71.5%)	1031 (1.7%)
	F	0	23,908 (28.5%)	430 (1.8%)

* Original TASH only considered BP < 100. Legend: TASH: Trauma-Associated Severe Hemorrhage; MT: massive transfusion; FAST: Focused Assessment with Sonography for Trauma; AIS: Abbreviated Injury Scale; M: male; F: female.

**Table 2:** Comparison between multiple surveyed categories between the ROTEM^®^ and non-ROTEM^®^ group.

	Non-ROTEM^®^ n = 76,058	ROTEM^®^ n = 7740	*p*-Value
Age	51.9 (21.0)	50.2 (20.5)	<0.001
Male patients	54,202 (71.3%)	5688 (73.5%)	<0.001
Mechanism: traffic	39,572 (52.6%)	4024 (52.5%)	<0.001
high fall	28,436 (37.8%)	2727 (35.6%)	
low fall	7168 (9.5%)	919 (12.0%)	
Injury Severity Score	19.7 (12.3)	24.3 (14.1)	<0.001
Penetrating trauma	3172 (4.4%)	425 (5.7%)	<0.001
Head injury (AIS 3+)	29,040 (38.2%)	3381 (43.7%)	<0.001
Thoracic trauma (AIS 3+)	31,273 (41.1%)	3633 (46.9%)	<0.001
Abdominal trauma (AIS 3+)	7860 (10.3%)	1280 (16.5%)	<0.001
Injury of the extremities (AIS 3+)	19,190 (25.2%)	2649 (34.2%)	<0.001
Quick’s value (%)	86 (22)	79 (23)	<0.001
INR	1.19 (0.55)	1.26 (0.61)	<0.001
Base excess (mmol/L)	−1.7 (4.5)	−3.0 (5.1)	<0.001
Hemoglobin (g/dL)	13.0 (2.2)	12.4 (2.5)	<0.001
Blood transfusion	7452 (9.8%)	1768 (22.2)	<0.001
Massive transfusion (10+ pRBC)	1168 (1.5%)	293 (3.8%)	<0.001
Length of stay on ICU (days)	3 (1–8)	5 (2–14)	<0.001
Length of stay in hospital (days)	12 (6–14)	16 (8–28)	<0.001
Time from accident to hospital admission (min)	69 (33)	73 (32)	<0.001
Admitted to level 1 hospital	63,315 (83.2)	7076 (91.4)	<0.001
Pre-hospital volume > 1000 mL	10,906 (15.4)	1508 (20.5)	<0.001
Died in hospital	9025 (11.9%)	1220 (15.8%)	<0.001
Expected mortality based on RISC II	11.3%	16.0%	<0.001
Standardized Mortality Ratio (SMR)	1.05 (1.03–1.07)	0.98 (0.93–1.04)	0.053

Values are indicated as mean or median with IQR. Legend: ROTEM^®^: rotational thromboelastometry; AIS: Abbreviated Injury Scale; ICU: Intensive Care Unit; RISCII: Revised Injury Severity Classification, version II.

## Data Availability

The data set used and analyzed during the current study is not available due to the data protection guidelines of the German Trauma Society.

## Supplementary Materials

The following supporting information can be downloaded at: https://www.mdpi.com/article/10.3390/jcm13144059/s1, Table S1: Logistic regression analysis of TASH components with massive transfusion as dependent variable.

## Abbreviations

ABC: Assessment of Blood Consumption, AIS: Abbreviated injury scale, AUC: Akademie der Unfallchirurgie GmbH, DGU^®^: Deutsche Gesellschaft für Unfallchirurgie, ICU: Intensive care unit, INR: International Normalized Ratio, ISS: Injury Severity Score, MT: Massive transfusion, NNT: Number needed to treat, POC: Point-of-care, PCC: Prothrombin complex concentrate, pRBCs: packed red blood cells, PT: Prothrombin time, RISC II: Revised Injury Severity Classification II, ROTEM^®^: Rotationsthromboelastometrie, SBP: Systolic blood pressure, SD: standard deviation, SMR: Standardized mortality ratio, TASH: Trauma-associated severe hemorrhage, TR-DGU^®^: TraumaRegister DGU^®^.
