# Supplementary material for: Early Point-of-Care Thromboelastometry Reduces Mortality in Patients with Severe Trauma and Risk of Transfusion: An Analysis Based on the TraumaRegister DGU®
Source: J Clin Med. 2024 Jul 11;13(14):4059. doi: 10.3390/jcm13144059 (PMC11277494; doi:10.3390/jcm13144059)
Supplement: Supplementary file 1 [file jcm-13-04059-s001.zip › jcm-2998486-supplementary.pdf]

Journal of
Clinical Medicine

MDPI

Table S1: Logistic regression analysis of TASH components with massive transfusion as dependent variable

	Regression coefficient	Standard error	Sig.	Odds ratio	95% confidence interval upper limit	lower limit
Hb (12+)			<.001			
Hb (<12)	1.061	0.111	<.001	2.889	2.324	3.592
Hb (<11)	1.247	0.112	<.001	3.480	2.793	4.335
Hb (<10)	1.607	0.112	<.001	4.987	4.003	6.212
Hb (<9)	2.037	0.100	<.001	7.669	6.299	9.336
Hb (<7)	2.588	0.108	<.001	13.308	10.777	16.434
BE (≥-2)			<.001			
BE (<-2)	0.744	0.094	<.001	2.105	1.750	2.531
BE (<-6)	1.237	0.101	<.001	3.446	2.829	4.199
BE (<-10)	1.715	0.097	<.001	5.559	4.596	6.722
BP (120+)			<.001			
BP (<120)	0.537	0.090	<.001	1.711	1.435	2.040
BP (<100)	0.756	0.109	<.001	2.129	1.718	2.638
BP (<90)	1.166	0.081	<.001	3.209	2.738	3.762
HR (>120)	0.589	0.072	<.001	1.802	1.564	2.077
FAST pos.	1.311	0.062	<.001	3.710	3.286	4.188
Instable pelvis	1.024	0.085	<.001	2.783	2.356	3.287
Femur fracture	0.647	0.063	<.001	1.909	1.687	2.160
Male	0.380	0.067	<.001	1.463	1.283	1.668
Constant	-7.101	0.109	<.001	0.001		
